# Isolation and Identification of Postharvest Rot Pathogens in *Citrus* × *tangelo* and Their Potential Inhibition with Acidic Electrolyzed Water

**DOI:** 10.1007/s12560-024-09604-4

**Published:** 2024-06-06

**Authors:** Ying Ji, Jieqiong Wang, Ye Liu, Shaoyan Liu, Xuanjing Jiang, Huaming Huang

**Affiliations:** 1 Fujian Forestry Vocational Technical College, Nanping, 353000 China; 2 Key Laboratory of Tea Biology and Resources Utilization, Ministry of Agriculture, Tea Research Institute Chinese Academy of Agricultural Sciences, 9 South Meiling Road, Hangzhou, 310008 China; 3 College of Oceanology and Food Science, Quanzhou Normal University, Quanzhou, 362000 China

**Keywords:** *Citrus* × *tangelo*, Fungal disease, Isolation and identification, Acidic electrolyzed water (AEW)

## Abstract

This study focused on the identification of rot-causing fungi in *Citrus* × *tangelo* (tangelo) with a particular emphasis on investigating the inhibitory effects of acidic electrolyzed water on the identified pathogens. The dominant strains responsible for postharvest decay were isolated from infected tangelo fruits and characterized through morphological observation, molecular identification, and pathogenicity detection. Two strains were isolated from postharvest diseased tangelo fruits, cultured and morphologically characterized, and had their gene fragments amplified using primers ITS1 and ITS4. The results revealed the rDNA-ITS sequence of two dominant pathogens were 100% homologous with those of *Penicillium citrinum* and *Aspergillus sydowii*. These isolated fungi were confirmed to induce tangelo disease, and subsequent re-isolation validated their consistency with the inoculum. Antifungal tests demonstrated that acidic electrolyzed water (AEW) exhibited a potent inhibitory effect on *P. citrinum* and *A. sydowii*, with EC_50_ values of 85.4 μg/mL and 60.12 μg/mL, respectively. The inhibition zones of 150 μg/mL AEW to 2 kinds of pathogenic fungi were over 75 mm in diameter. Furthermore, treatment with AEW resulted in morphological changes such as bending and shrinking of the fungal hyphae surface. In addition, extracellular pH, conductivity, and absorbance at 260 nm of the fungi hypha significantly increased post-treatment with AEW. Pathogenic morphology and IST sequencing analysis confirmed *P. citrinum* and *A. sydowii* as the primary pathogenic fungi, with their growth effectively inhibited by AEW.

## Introduction

*Citrus* × *tangelo* (tangelo) is a modern Japanese hybrid citrus cultivar developed from a cross between the female *Citrus unshiu* (mandarin) and the male *Citrus hassaku* (Hassaku orange) (Ji et al., 2022). Tangelo is distinguished by its orange–yellow color, rough skin, and oblate shape. Its pulp is orange-red, tender, and juicy, making it a widely consumed fruit. However, its susceptibility to minor injuries during harvesting and transportation renders it prone to pathogen infection during storage. Such infections lead to fruit softening, rotting, and the development of an unpleasant odor, ultimately diminishing its commercial value. Therefore, elucidating the pathogens responsible for postharvest diseases in tangelo and seeking efficient, environmentally friendly, and safe storage and preservation methods have become key challenges in advancement of the tangelo industry.

While extensive research has been conducted on microbial diseases affecting citrus fruits (Beatriz et al., 2019; Bonants et al., 2003; Martina et al., 2022; Mondal et al., 2012; Yogi et al., 2022), little attention has been given to the isolation and identification of the primary decay-causing fungi in tangelos, postharvest. Despite acquiring scab and ulcer disease resistance from the *Hassaku* orange and the *Citrus unshiu*, respectively, tangelos remain susceptible to anthracnose (Douanala-Meli & Unger, 2017; Mayorquin et al., 2019; Uysal & Kurt, 2019). Conventional chemical fungicides are commonly used for controlling these fungal diseases, yet pose challenges such as drug residue and environmental pollution.

Electrolytic water, also known as electrogenerated functional water, is the product of sodium chloride solution or dilute hydrochloric acid after electrolysis in an electrolytic system, and it can be divided into three types, including acidic electrolyzed water (AEW), slightly acidic electrolyzed water (SAEW), and alkaline electrolyzed water (ALEW). Generally, AEW has a low pH (< 2.8), high redox potential or oxidation–reduction poten_tial (ORP) (ORP > 1050 mV) and high available chlorine concen_tration (ACC, greater than 5 mg/L) (Chen et al., 2017).

In recent years, AEW has emerged as an environment-friendly and emerging antimicrobial sanitizer, owing to its safe, minimal pollutant, non-residue, non-toxic, simple formulation, and cost-effective properties (Chen et al., 2020; Hu et al., 2019; Puglisi et al., 2017).AEW has been used to mitigate the fungal infections on fruits surface during postharvest storage and handling. Studies by Youssef et al. highlighted the effectiveness of AEW in controlling *penicillium* in sweet oranges (Hyun et al., 2019), while Sierra et al. reported its potent inhibitory effect on mold in strawberries (Youssef & Hussien, 2020). However, to our knowledge, the effect of AEW as a postharvest treatment on fruit quality particularly regarding postharvest rot pathogens of tangelo has not been reported.

The precise causes of postharvest rot in tangelo fruits remain unclear, hindering timely prevention and control measures. This study aimed to isolate and purify pathogenic fungi from postharvest diseased tangelo fruits, validate their pathogenicity through inoculation test, and use molecular techniques to identify the dominant fungi responsible for postharvest rot disease. Concurrently, AEW was subjected to in-vitro antifungal experiments, and antifungal activity was screened through indoor toxicity evaluation. The optimal concentration of AEW was selected for preliminary exploration of its antifungal efficacy.

## Materials and Methods

### Materials

Naturally diseased apples: 65 diseased apples were randomly collected from plantations in Nanping City, Fujian province from October to November, 2022.

Vaccination materials: The diseased and healthy fruits were collected from the tangelo storage and fresh-keeping training base of Fujian Forestry Vocational Technical College.

Preparation of AEW: In order to produce the AEW, 0.03 g/100 mL NaCl solution was electrolyzed using hypochlorite water generator from Weyhill Environmental Technology (Shanghai, China) Co. Ltd. The pH of AEW was determined with a pH meter (PHS-3E, Shanghai, China), Iodometry method (Jiao et al., 2023; Pang et al., 2023) was used to measure the available chlorinemass concentration (ACC) of AEW. The initial ACC of AEW was 300 mg/L. The concentration required for subsequent tests was diluted with 300 μg/mL AEW in water, and pH of AEW was adjusted to 2.5 ± 0.1 using 0.1 mol/L HCl.

### Isolation and Purification of Pathogenic Fungi

The pathogens were isolated from diseased samples using the tissue isolation method (Daoud et al., 2019; Guarnaccia & Crous, 2017). Tissues, approximately 5 mm × 5 mm in size, were excised with a sterile scalpel, rinsed three times in sterile water, soaked in 75% alcohol for 20 s, and then treated with 0.1% mercuric chloride for 2 min. The tissues were then rinsed three times in sterile water, excess surface water was removed with sterile filter paper, transferred to potato dextrose agar (PDA), and incubated at 28 °C. Once hyphae were visible, they were cultured on PDA until a pure fungal strain was obtained. The pure fungal strain was then identified based on morphological characteristics and stored at 4 °C in test tubes containing the inclined plane of PDA for further use (Quang et al., 2023).

### Morphological Observation of Pathogenic Fungi

The purified pathogens were inoculated on new PDA plates and incubated at 28 °C to observe colony characteristics. Simultaneously, colony morphology, hyphae size, and spore size were observed with a biological microscope (E200, Japan).

### Molecular Identification of Pathogenic Fungi

The DNA extraction followed a previously described method (Bui et al., 2022) with minor modifications using the Ezup column fungal genomic DNA extraction kit (Sk 8259) (Sangon Biotech Co., Ltd, Shanghai, China). Amplification of DNA was carried out using thermocycler with universal primers ITS1 (TCCGTAGGTGAACCTGCGG), ITS4 (TCCTCCGCTTATTGATATGC), NS1 (GTAGTCATATGCTTGTCTC), and NS6 (GCATCACAGACCTGTTATTGCCTC). The Polymerase Chain Reaction (PCR) reaction was set as follows: Template (genomic DNA 20–50 ng/μL) 0.5 μL, 10 × Buffer (with Mg^2+^) 2.5 μL, dNTP (2.5 mM) 1 μL, enzyme 0.2 μL, Forward primer (10 μM) 0.5 μL, Reverse primer (10 μM) 0.5 μL, distilled water to 25 μL; and the PCR cycle conditions as follows: 94 °C pre-denaturation for 4 min, 94 °C denaturation for 45 s, 55 °C annealing for 45 s, 72 °C extension for 1 min, 30 cycles, 72 °C final extension for 10 min, 4 °C preservation. The PCR products were analyzed by gel electrophoresis with 1% agarose. Subsequently, the PCR products were sent to (Sangon Biotech Co., Ltd, Shanghai, China) for sequencing, and the sequencing results were searched against the NCBI nucleotide database using the sequence alignment tool BLASTn. The phylogenetic tree was constructed using ITS gene sequences of homologous and related species in MEGA7 (Nakarin et al., 2022; Rajeendran et al., 2017).

### Pathogenicity Test of Isolated Fungi

Healthy fruits with uniform size, color, and no mechanical damage were selected. Fruit surfaces were disinfected with 75% alcohol, washed with sterile water, and dried with sterile filter paper. A 5 mm diameter fungal cake was obtained from the edge of a fresh colony (cultured on a PDA plate for 5 days) using a sterile perforator. Two inoculation methods were employed: (1) Puncture inoculation: a 5 mm deep hole was punctured on the citrus fruit using a sterile needle, and a fresh fungal cake inoculated in the puncture with the fruit surface upside down (Tamura et al., 2013). (2) Inoculation without injury: a fresh fungal cake was inoculated on the fruit surface upside down (Zhang et al., 2022). Control tests used a 5 mm diameter PDA agar block inoculated upside down on the fruit surface. Inoculated samples were individually placed in microporous bags and cultured in five replicates at 28 °C. The pathogenic fungi were isolated and verified by Koch’s rule (Jiang et al., 2022).

### Inhibition of AEW to Pathogenic Fungi

In vivo test: AEW treatment and inoculation of *Penicillium citrinum* and *Aspergillus sydowii.*

(1) Toxicity test in the laboratory: The inhibitory effect of AEW at different concentrations on pathogenic fungi was evaluated by the Oxford cup method. Fresh pure cultures were mixed in 0.9% NaCl solution, cultured at 28 °C for 24 h, and the suspension was mixed into PDA medium which was cooled to 46 °C at a ratio of 1:9 to make a plate containing fungi. Oxford cups were placed in the center of the plates, and 200 μL of AEW (50, 100, 150, 200, 250 μg/mL) was added. The plates were cultured at 28 °C for 3 days, with sterile water as a control.

Anti-fungal activity rate was calculated as follows:

$$IR = \frac{{D_{2} }}{{D_{0} - D_{1} }} \times 100\%$$

where IR is the anti-fungal activity rate, *D*_0_ is the diameter of the dish, *D*_1_ is the average diameter of the anti-fungal zone in the control group, and *D*_2_ is the average diameter of the anti-fungal zone in the treatment group.

Statistical analysis of the data using SPSS. 20, using least squares, established a linear equation for “Logarithmic-probability value of mass concentration,” where *x* was the logarithmic value corresponding to the AEW concentration, *y* was the probability value of antifungal activity conversion rate. The toxicity regression equation of AEW was obtained, and the effective medium concentration of AEW (EC_50_) and the calculation of linear correlation coefficients (R^2^).

Evaluation of fruit diseases: The tangelo fruits were soaked in AEW with EC_50_ for 20 min, and then inoculated with *P. citrinum* and *A. sydowii* spore suspension after drying. The fruits were kept to 90% relative humidity and 28 °C. Forty tangelos were sampled to assess the fruit disease index referring to the methodologies of Tang et al. (2021). According to the ratio of the lesion area on the surface of fruit to the total area of forty individual tangelo fruit, the severity of tangelo fruit disease consists of five types, 0 type fruit: No Lesion, 1 type fruit: Lesion area < 1/4, 2 type fruit: 1/4 ≤ Lesion area < 1/2, 3 type fruit: 1/2 ≤ Lesion area < 3/4, 4 type fruit: Lesion area ≥ 3/4. The index of tangelo fruit disease was calculated as Σ (disease type/the highest type × percentage of corresponding tangelo fruit in each type).

In vitro test: The effect of AEW treatment and inoculation of *P. citrinum* and *A. sydowii.*

1. Determination of the changes in the fungal body: Fungal cakes were prepared from well-growing cultures after 5 days of incubation, inoculated in AEW at EC_50_, and cultured at 28 °C for 24 h. After washing with aseptic water three times, the fungi were air-dried and observed with the microscope (E200, Japan).
2. The determination of extracellular pH value: The pathogens were inoculated in Potato dextrose broth (PDB) medium, cultured at 28 °C for 48 h, shaking 160 times/min. (HY-2, China). The cultures were washed with sterile water and centrifuged at 5000 rpm, 3773×*g* (H1850, China) for 20 min (Han et al., 2022). The precipitated mycelium was mixed in a Phosphate buffered saline (PBS, pH 7.0) buffer and treated with AEW at EC_50_ for 0, 30, 60, 90, and 120 min. Extracellular pH values with a pH meter (PHS-SE, China) were determined three times, with PBS buffer as a control.
3. Determination of extracellular conductivity: The method of culture treatment in this experiment was the same as in test (2). The extracellular conductivity of the treatment solution measured three times with electrical conductivity meter (DDS-11A, China).
4. Cell release assay: The method of culture treatment in this experiment was the same as in test (2). The sample was centrifuged at 11,000 rpm, 12,840×*g* (TG16-WS, China) for 5 min, the supernatant was collected, and absorbance was determined three times at 260 nm with Ultraviolet photo meter(TU-1810PC, China).

### Statistical Analyses

The whole experiment was designed following a randomized experiment design with triplicate samples collected on each sampling date. Data are presented as means standard errors. Analysis of variance was conducted using SPSS statistics tool version 20.0, with *p* < 0.05 considered significant and *p* < 0.01 very significant. The WPS software was used for graph plotting.

## Results

### Morphological Characteristics of Pathogenic Fungi

According to the symptoms of 65 diseased tangelo fruits, the pathogen was isolated from the lesions with different symptoms. A total of 20 strains were isolated. According to the colony color, colony growth rate and colony morphology, the strains were classified into 3 groups. 11(55%) strains were classified as Class I, 8(40%) strains were classified as Class II, two strains of Class III were isolated, accounting for 5%. Therefore, it is inferred that Class I and Class II strains are the dominant pathogens, named as TF1 and TF2, respectively. According to the morphological characteristics of the pathogen, it was identified as fungi. In the follow-up of this experiment, the dominant bacteria of 2 strains were identified.

**Fig. 1:**
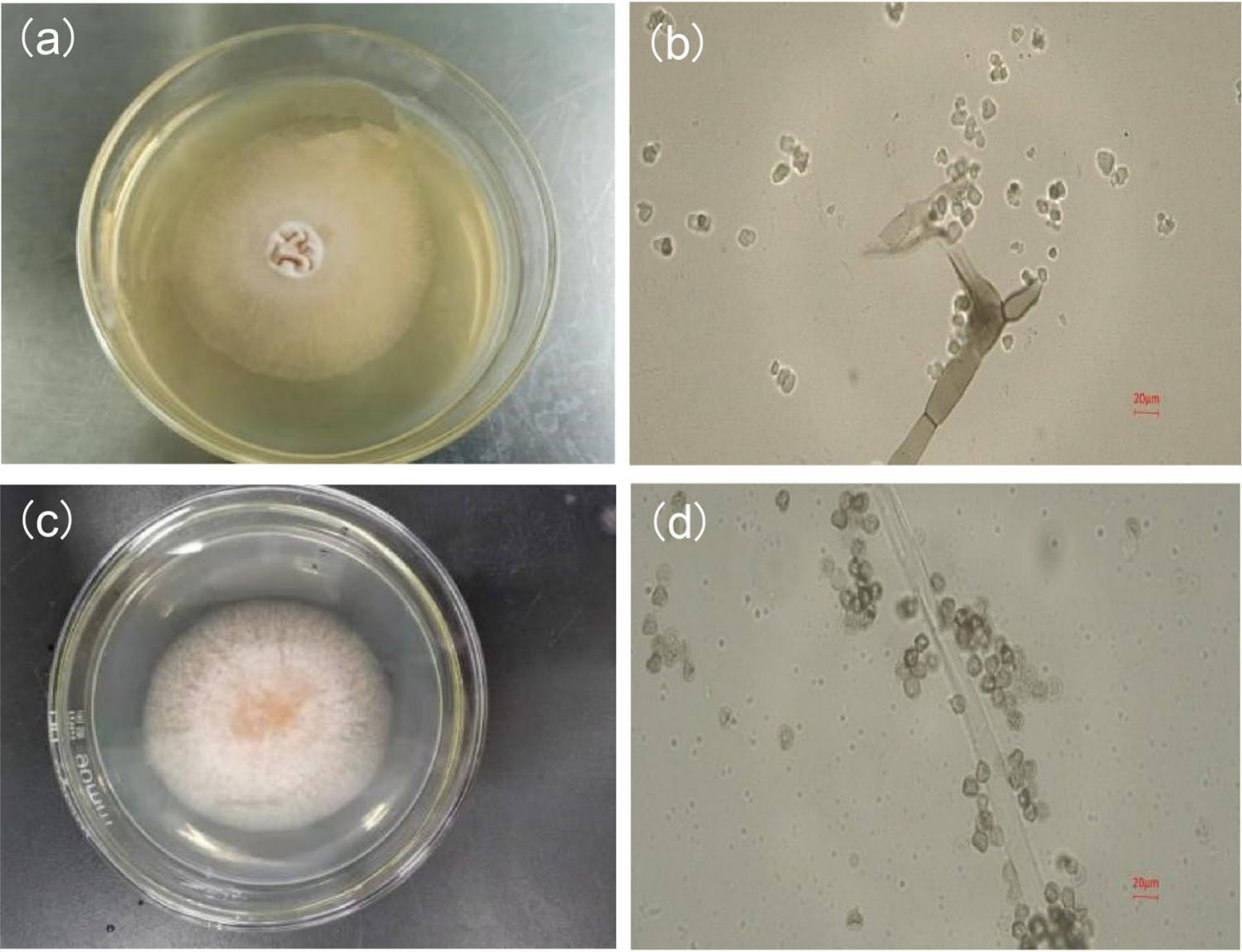
Morphological characteristics of pathogenic fungi. **a** Cultures of strain TF1 on PDA. **b** The mycelium and conidia of strain TF1. **c** Cultures of strain TF2 on PDA. **d** The mycelium and conidia of strain TF2

TF1 exhibited a petaline-like velveteen bulge at the center of the colony on PDA, with a light pink outer ring displaying a large area of dense white short-haired hyphae (Fig. 1a). Microscopic examination revealed erect conidia with a few branches, septa, and a light brown, stubby appearance, displaying various shapes, including rounded and square (Fig. 1b). The colony surface of strain TF2 on PDA was fluffy, with dense short villi, white, and a yellowish center (Fig. 1c). Microscopic observation showed that the conidia of the fungus had no septa, were long strip-shaped, with dense conidia piles around them, appearing brown, transparent, round or nearly round (Fig. 1d).

### Molecular Biological Identification of Pathogenic Fungi

**Fig. 2:**
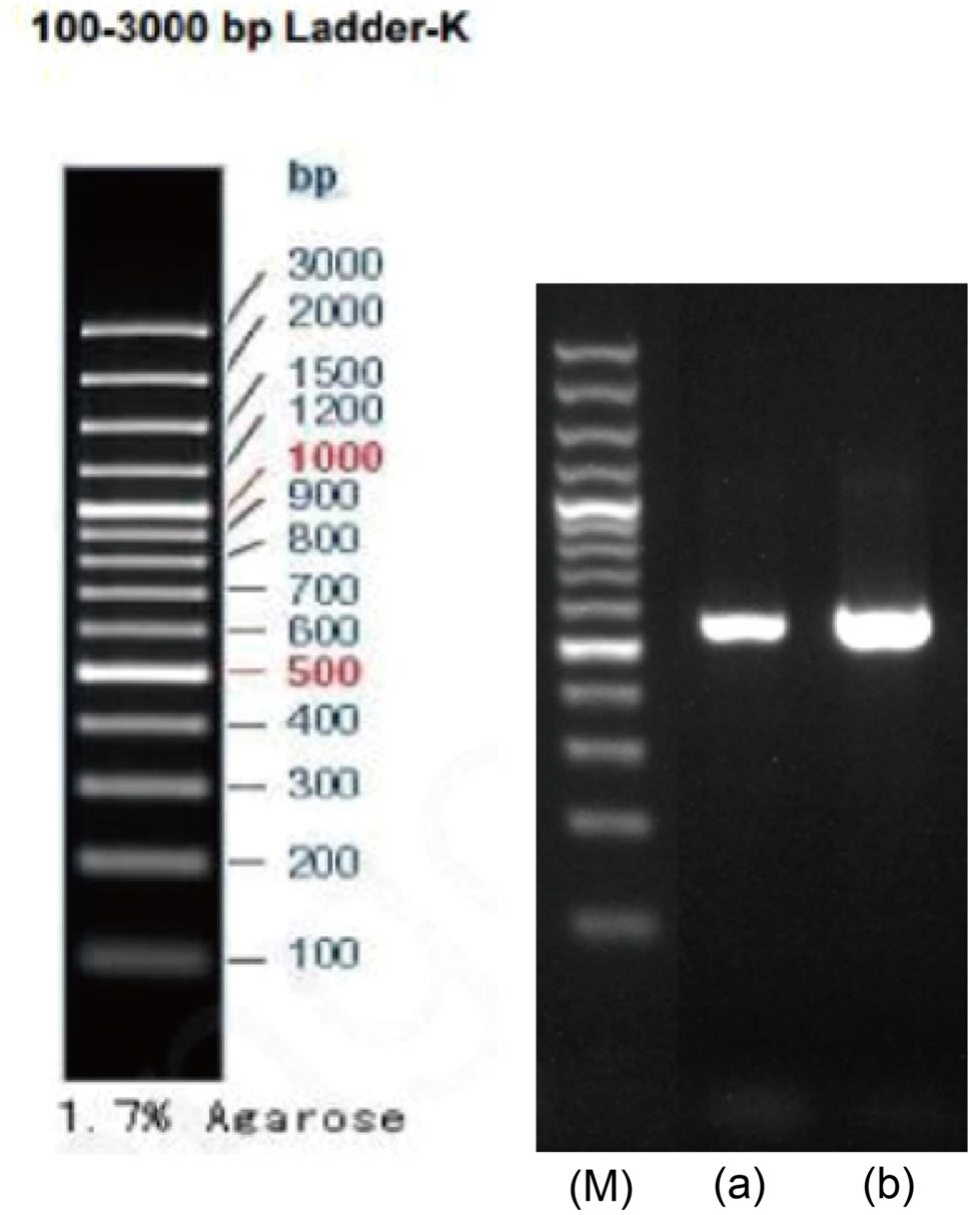
Gel electrophoretic assay of the pathogenic fungi PCR fragments. (M), (a), and (b) represent Marker DL 2000, TF1, and TF2 amplification products, respectively

**Fig. 3:**
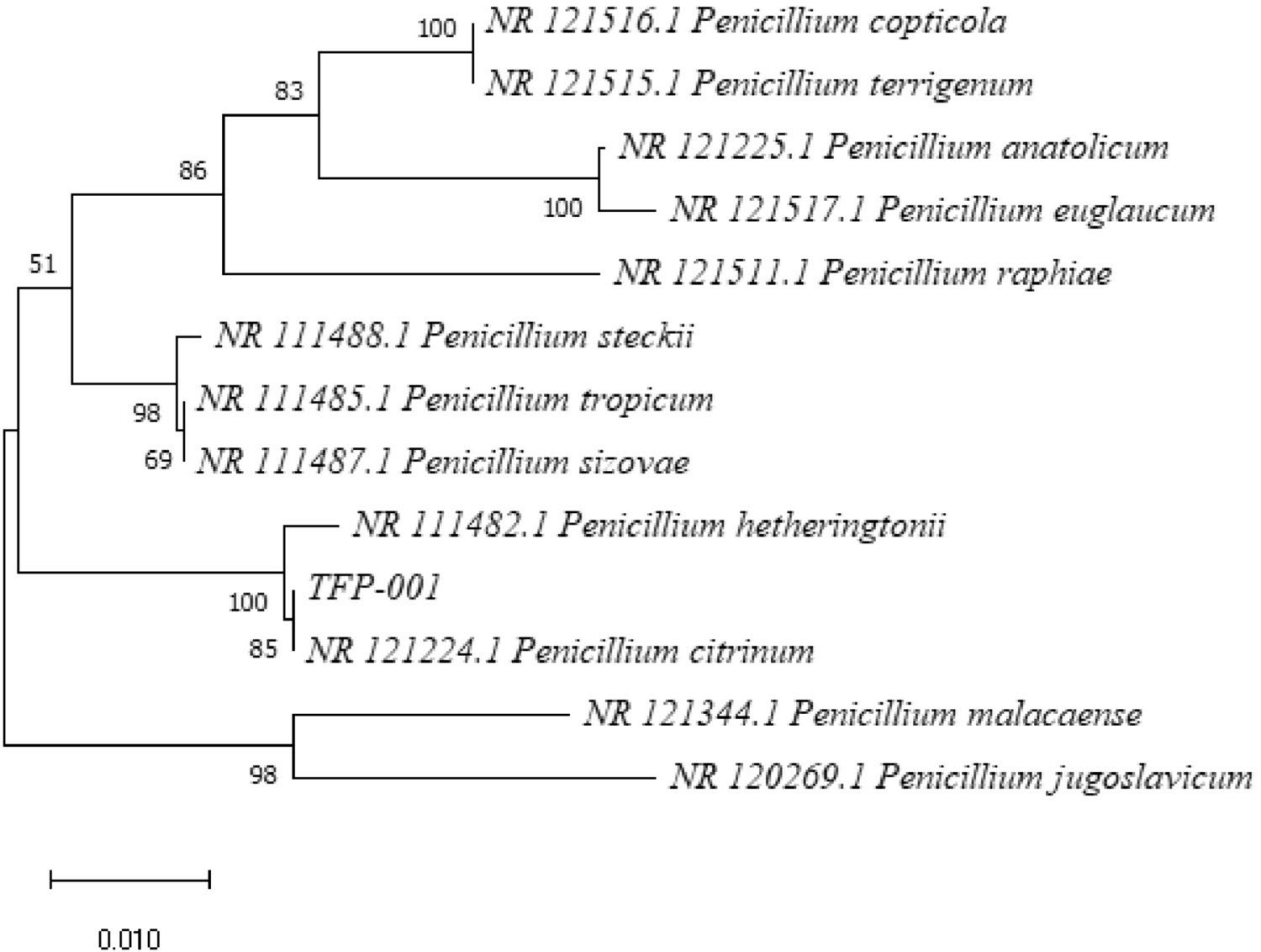
Phylogenetic tree based on ITS sequences of strain TF1

**Fig. 4:**
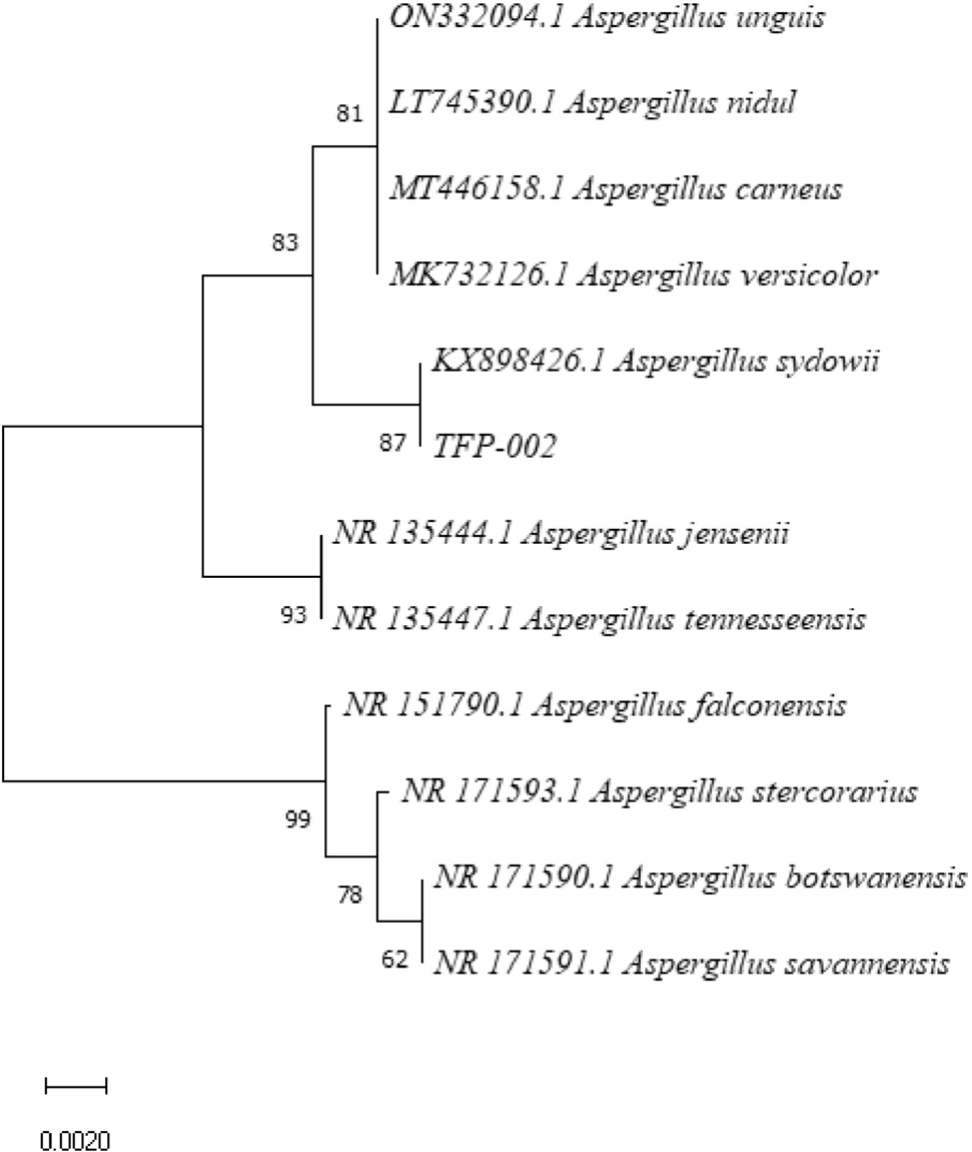
Phylogenetic tree based on ITS sequences of strain TF2

The PCR products were analyzed by agarose gel electrophoresis, revealing amplified fragments of 530 bp for TF1 and 546 bp for TF2 (Fig. 2). The r-DNA-ITS sequences of strains TF1 and TF2 were submitted to GenBank (Accession Numbers: OP526939.1 and OP103947.1, respectively). The BLAST results indicated a 100% homology between strain TF1 and *P. citrinum* and between strain TF2 and *A. sydowii* (Table 1). Phylogenetic trees constructed by homologous sequences confirmed the evolutionary positions of TF1 and TF2 (Figs. 3 and 4).

**Table 1:** The sequence identification information of pathogenic fungi was carried out on NCBI

Numbei	Description	Max score	Total score	Query cover	Evalue	Per. Ident	Accession
TF1	*P. citrinum* isolate Pci0001 small subunit ribosomal RNA gene, partial sequence; internal transcribed spacer 1, 5.8S ribosomal RNA gene, and internal transcribed spacer 2, complete sequence; and large subunit ribosomal RNA gene, partial sequence	979	979	100%	0.0	100.00%	OP526939.1
TF2	*A. sydowii* strain C58N small subunit ribosomal RNA gene, partial sequence; internal transcribed spacer 1, 5.8S ribosomal RNA gene, and internal transcribed spacer 2, complete sequence; and large subunit ribosomal RNA gene, partial sequence	1009	1009	100%	0.0	100.00%	OP103947.1

In summary, morphological identification, NCBI comparison, and phylogenetic tree analysis identified strain TF1 as *P. citrinum* and strain TF2 as *A. sydowii*.

### Pathogenicity Test of Pathogenic Fungi

**Fig. 5:**
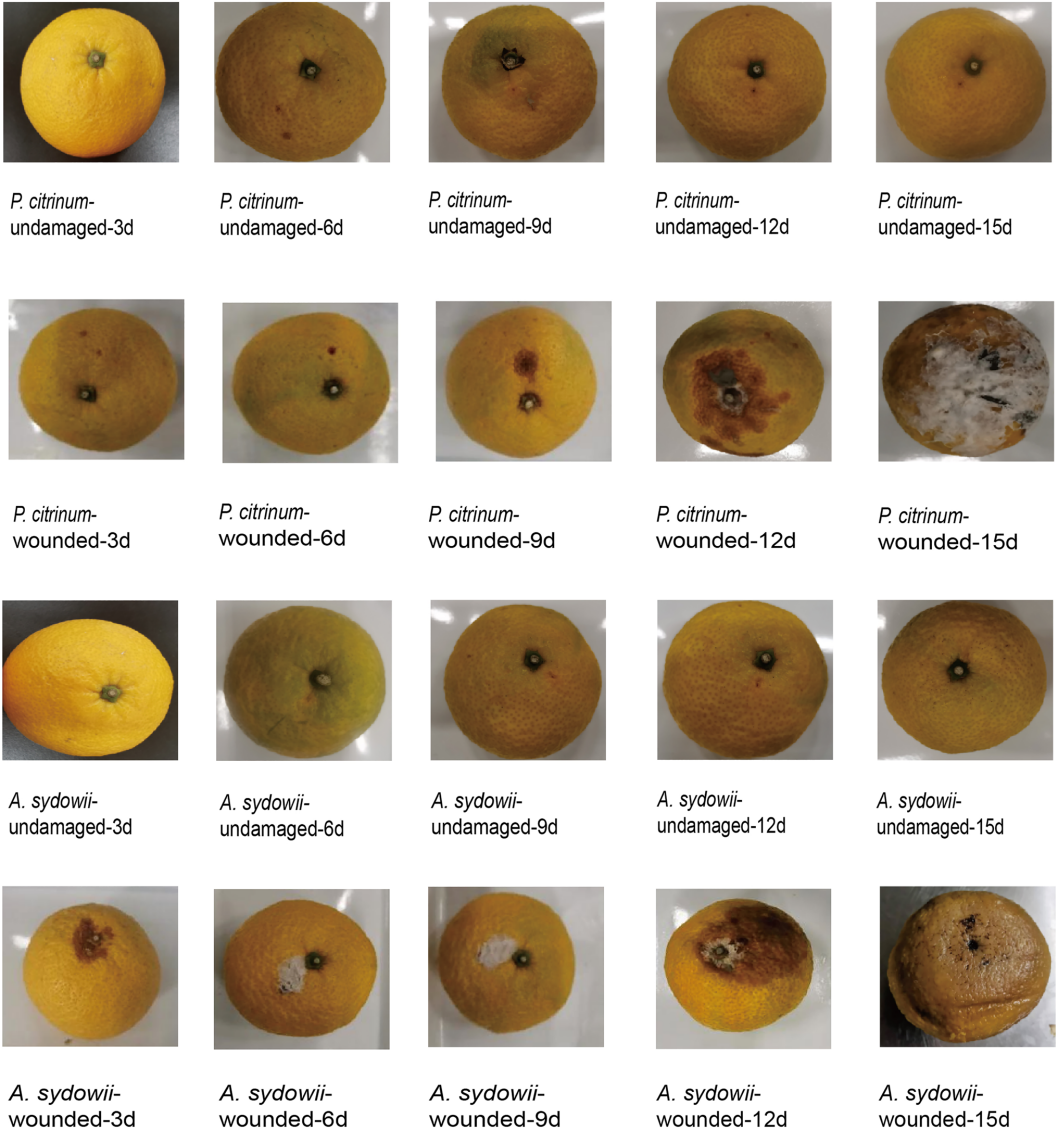
Symptoms of tangelo fruits inoculated with two kinds of pathogens

Figure 5 showed that inoculation of the strains into healthy mature fruits resulted in observable symptoms at 3, 6, 9, 12, and 15 days. The symptoms on fruit-undamaged were not obvious until the 12th day, with peduncles shrinking followed by fruit softening. Inoculation test of *P. citrinum* showed the skin softened on the third day, lesions appeared on the sixth day, and decay as well as white mold formation occurred by the fifteenth day. Inoculation test of *A. sydowii* showed the fruit induced lesion on the third day, hyphae appearance and fruit softening occurred on the sixth day, and extensive decay with juice outflow occurred by the fifteenth day. The morphological characteristics of the inoculated fruits were consistent with those of the original pathogen.

### The Toxicity of AEW to Pathogenic Fungi in the Laboratory

AEW exhibited inhibitory effects on *P. citrinum* and *A. sydowii*, with varying efficacy across different concentrations. The EC_50_ values of *P. citrinum* and *A. sydowii* were 85.4 μg/mL and 60.12 μg/mL, respectively. Strain *A. sydowii* demonstrated greater sensitivity to AEW than *P. citrinum* (Table 2).

**Table 2:** The results of toxicity test of AEW to pathogenic fungi

Pathogen	AEW (μg/mL)	Average diameter of bacteriostatic ring (mm)	Bacteriostasis rate (%)	Toxicity regression equation	R^2^	EC_50_ (μg/mL)
*P. citrinum*	50	11.83 ± 1.82a	13.6	*y* = 4.7172 *x*- 4.1111	0.9391	85.4
	100	24.55 ± 2.13b	62.7			
	150	77.34 ± 2.84c	88.9			
	200	82.13 ± 2.61d	94.4			
	250	86.04 ± 3.85e	98.9			
*A. sydowii*	50	35.58 ± 1.87a	40.9	*y* = 2.8828 *x*- 0.1272	0.9270	60.12
	100	67.68 ± 2.76b	77.8			
	150	76.04 ± 2.54c	87.4			
	200	81.34 ± 3.12d	93.5			
	250	83.78 ± 3.44e	96.3			

Mean ± SD is shown (*n* = 3), and distinct letters in the table indicate statistically differences among different AEW treatments (*p* < 0.05)

### Effect of AEW Treatment on Disease Resistance of Tangelo Fruits

**Fig. 6:**
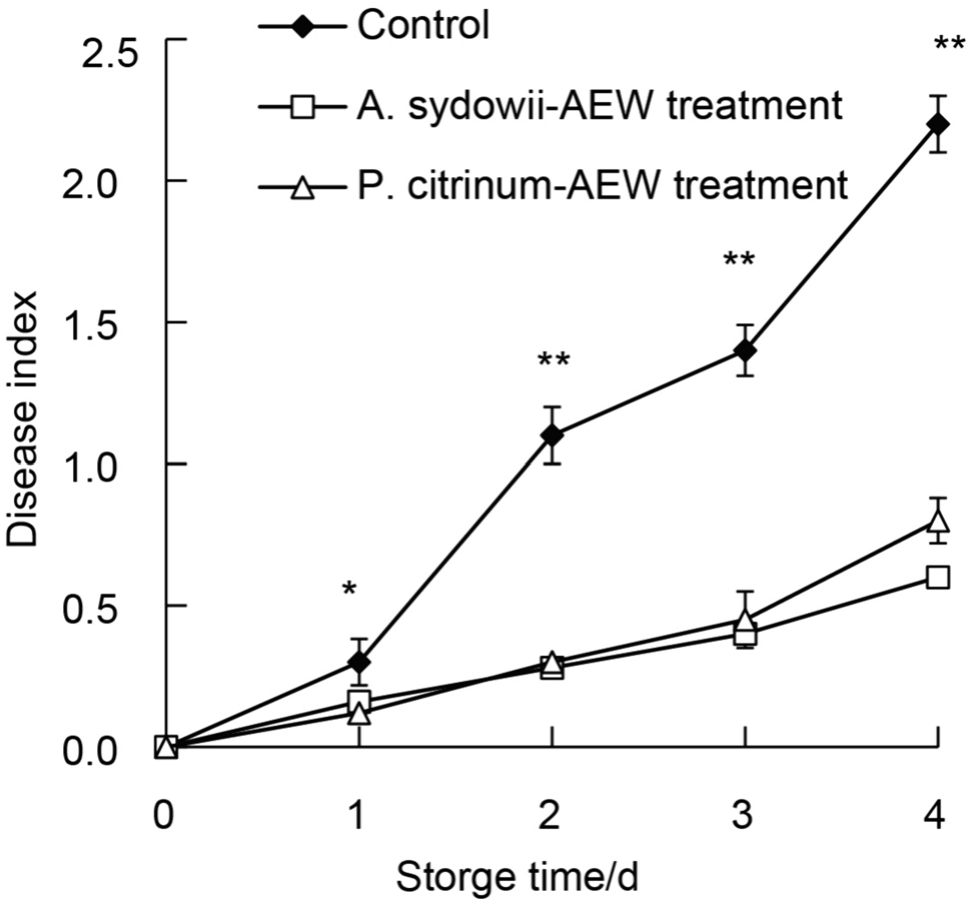
Effect of AEW (EC_50_) on tangelo fruits disease index. Values are the mean ± standard error (*n* = 3). Significant differences between are represented by * (*P* < 0.05) and ** (*P* < 0.01). (Filled diamond) control; (Open triangle) *P. citrinum*-AEW treated fruit; (Open square) *A. sydowii*-AEW treated fruit

Figure 6 showed that AEW-treated fruits had significantly lower disease indices on days 1 to 4 than control samples. These results indicated that AEW treatment could delay the occurrence of disease development and maintain a lower fruit disease index in comparison with the control samples.

### Effect of AEW on the Morphology of Pathogenic Fungi

**Fig. 7:**
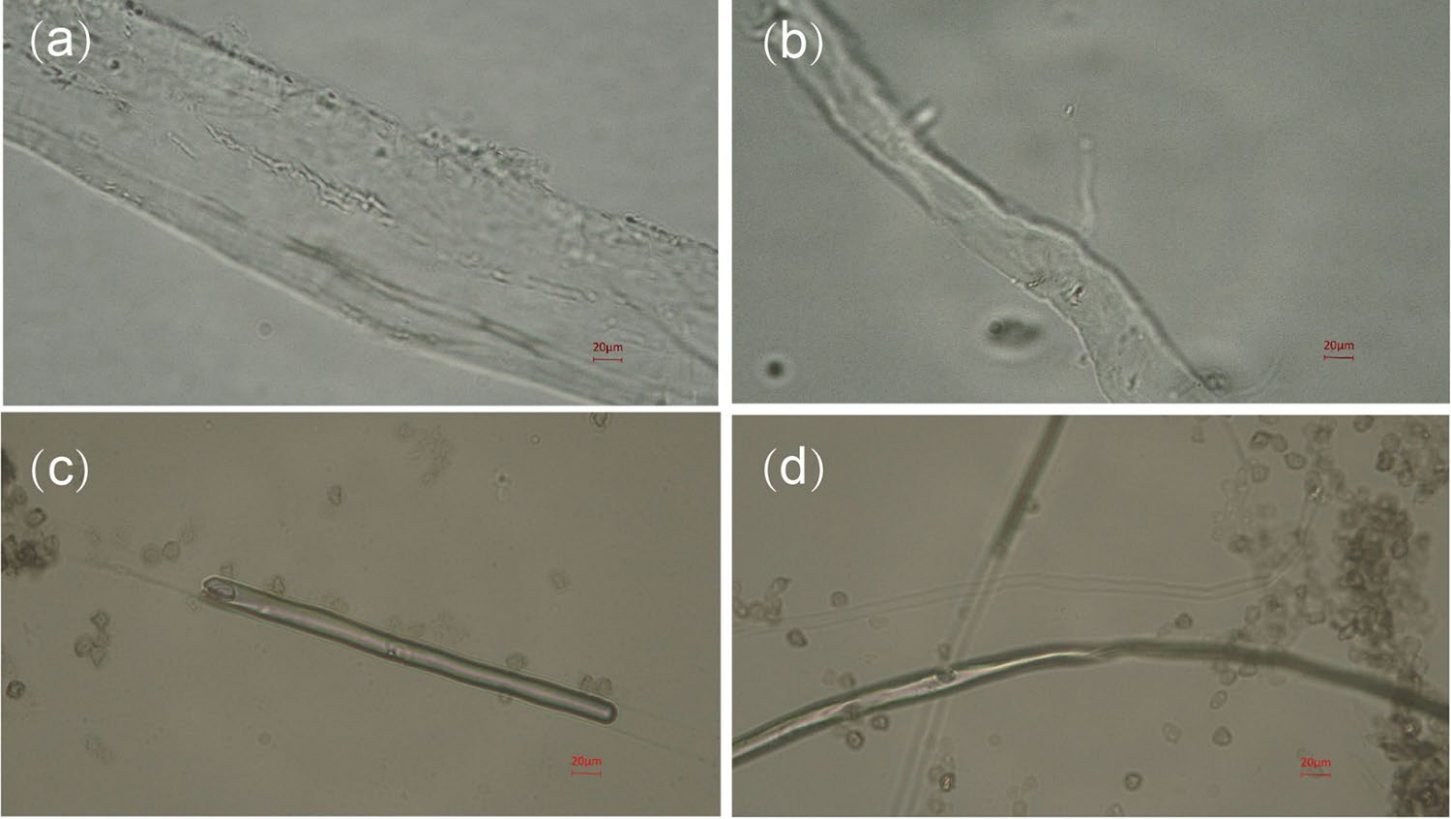
Effect of AEW on the morphology of pathogenic fungi. **a** *P. citrinum* of the control group. **b** *P. citrinum* was treated with AEW. **c** *A. sydowii* of the control group. **d** *A. sydowii* was treated with AEW

Microscopic examination showed that the surface of the mycelium of *P. citrinum* was stout, even, round and cylindrical in the control group (Fig. 7a). However, the surface of the mycelium of *P. citrinum* collapsed, distorted, and contracted after treatment with AEW (Fig. 7b). *A. sydowii* of the control group showed a flat, uniform, small cylindrical shape (Fig. 7c), while *A. sydowii* of the treatment showed obvious bending on the surface of the mycelium, and the ends of the mycelium appeared dry and small, the contents of the cells were significantly reduced (Fig. 7d). It was possible that AEW treatment caused leakage of intracellular substance of the mycelium and the destruction of its cell membrane integrity.

### Effect of AEW on Extracellular pH of Pathogenic Fungi

**Fig. 8:**
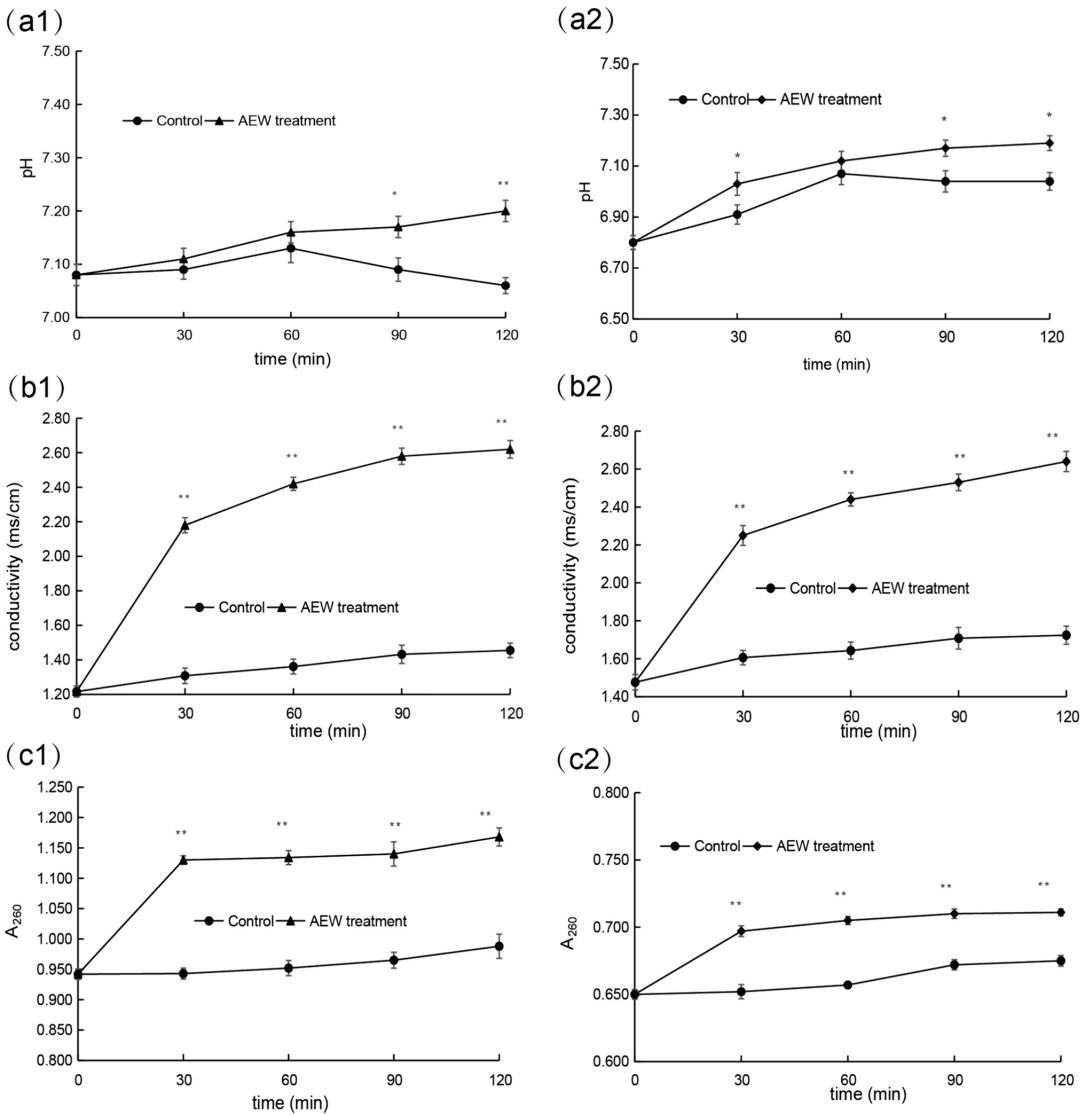
Effect of AEW on pathogenic fungal extracellular pH, conductivity, and the release of cellular components. (**a1**), (**b1**), and (**c1**) *P. citrinum* treated with AEW: (**a2**), (**b2**), and (**c2**) *A. sydowii* treated with AEW. Mean ± SD is shown (*n* = 3). Significant differences between are represented by * (*P* < 0.05) and ** (*P* < 0.01). (Filled diamond) AEW treatment; (Filled circle) control

pH controls DNA transcription, protein synthesis, and enzyme activity. When the treatment time was less than 60 min, there was no significant difference in extracellular pH between the control group and the treatment group. From 60 to 120 min, extracellular pH values increased significantly for both strains treated with AEW. TF1 and TF2 showed pH values of 7.20 and 7.19, respectively, at 120 min, significantly higher than that of the control group (*p* < 0.05). (Fig. 8a). The results showed that the treatment of AEW increased the extracellular pH of pathogenic fungi, which meant that the intracellular pH decreased. Intracellular acidification was caused by the accumulation of H^+^, which led to the permanent imbalance of intracellular pH, which led to cytoplasmic acidification, degeneration and energy loss (Oonmetta-aree et al., 2006).

### Effect of AEW on Extracellular Conductivity of Pathogenic Fungi

Abnormal increase of cell membrane permeability is the early manifestation of cell injury. Electrical conductivity in the treated group increased continuously, with the fastest increase in the first 30 min. *P. citrinum* and *A. sydowii* exhibited significantly higher electrical conductivity compared to the control group (*p* < 0.05). After the first 30 min, electrical conductivity continued to increase, with the increase for *P. citrinum* at 120 min being 1.165 ms/cm higher than that of the control and for *A. sydowii* being 0.916 ms/cm higher than the control (*p* < 0.05) (Fig. 8b). These results showed that AEW could destroy the cell membrane structure of fungi, increase the cell membrane permeability of *P. citrinum* and *A. sydowii*, and increase the extracellular conductivity of the fungi.

### Effect of AEW on the Release of Cell Components from Mycelium of Pathogenic Fungi

260 nm is the characteristic wavelength of nucleic acid. As indicated by a significant increase in A_260_ values in the treated group compared to the control group (*p* < 0.05). At 120 min, A_260_ values for *P. citrinum* and *A. sydowii* were 1.168 and 0.711, respectively, significantly higher than that of the control group (*p* < 0.05) (Fig. 8c). This indicated increased leakage of nucleic acid and other substances in the cells of pathogenic fungi, suggesting that the genetic process in the cells of pathogenic fungi was greatly affected by AEW.

## Discussion

### Study on the Main Pathogens of Post-harvest Rot of Tangelos

There were many reports on citrus fungal diseases (Chang et al., 2020; Guo & Hou, 2013; Pisani et al., 2015). *Penicillium digitatum* and *Penicillium italicum* were the most important sources of postharvest decay attacking citrus and late oranges (Martina et al., 2022; Youssef & Hussien, 2020). At present, there was little research on tangelos diseases. Tangelos diseases were mainly caused by *Anthracnose* and *Canker*, among which anthracnose is a serious disease (Ye, 2015). *Colletotrichum gloeosporioides* was a common fungal disease that infects the branches, leaves and fruits of plants. The rot of tangelo fruits caused by fungi was very common during the middle and late growth period, transportation and storage. This project mainly studied tangelo postharvest fruit rot.

In this study, we successfully isolated dominant strains from postharvest rot tangelo fruits. Through morphological characterization and molecular identification, it was determined that the pathogens causing postharvest diseases of tangelo were *P. citrinum* and *A. sydowii*. To fulfill Koch’s postulates, pathogenicity was tested for all strains that had developed the same symptoms as those observed in the field. Our findings were supported by those of previous studies which reported that *P. citrinum* and *A. sydowii* were economically important plant pathogen fungi and that *P. citrinum* and *A. sydowii* had been reported to cause various disease symptoms in citrus plants in tropical and subtropical regions (Guo et al., 2015; Ouyang et al., 2014; Tournas, 2005; Youssef & Hussien, 2020). In China, the causes of many post-harvest fruit diseases had been identified prior to this research. For examples, *Aspergillus niger* was the main pathogen of pomegranate fruit rot (Chang et al., 2020), *Geotrichum candidum* was the main pathogens of peach fruit rot (Zhang et al., 2022), *Rhizopus stolonife*r was the important pathogen causing rot of strawberry (Beatriz et al., 2019) and *Phomopsis longanae* was the most important pathogen causing rot of longan (Chen et al., 2014). However, there have been no prior reports of identification of postharvest pathogen of tangelo in China.

This study reveals that *P. citrinum* primarily infects tangelo fruits in the early storage period, easily invading through micro wounds. Initial symptoms include brown lesions on the fruit skin, followed by the appearance of white hyphae in the middle stage. As the disease progresses, lesions expand, accompanied by a white mold layer, resulting in festering, serous decay, and a moldy smell. In addition, we found that in the early stage of *A. sydowii* infection, citrus fruit surface will appear disease spots, with a mold layer, and then the fruit gradually soft rot. This was consistent with the symptoms observed in citrus experiments (Xie et al., 2013; Xu & Du, 2021). The prevalence of *A. sydowii* in tangelos cultivation regions, such as in Fujian, Zhejiang, and other places in China, could be facilitated by the subtropical monsoon climate, characterized by moderate temperature and abundant rainfall, especially during storage when the environment is suitable. Accurate identification of *P. citrinum* and *A. sydowii* provides a theoretical foundation for the prevention and control of postharvest rot disease in tangelos. However, due to the wide host range associated with twe fungis, they can produce pycnidia and release conidia that then accumulate in the atmosphere surrounding the plants as well as in the soil. Follow-up studies are needed to the distribution of twe pathogenic fungis in other tangelos plantations.

### Studies on the Inhibition of AEW to Two Kinds of Pathogenic Fungi

The pathogen of tangelo postharvest was isolated and identified in the early stage. Now we need to take an efficient approach.

AEW is widely used in food, agriculture, and medical fields because of its wide sterilization range, obvious effect, safety to human body and environment, convenient preparation and low cost. In 2002, Japan included AEW in the list of legal food additives. In 1994, AEW developed rapidly after it was introduced in China, and in 2020, the country promulgated GB 28234–2020 (Hygienic requirements for acid electrolytic water generator). AEW is a promising fungicide due to its efficiency, safety, and minimal pollution (Chen et al., 2019a, 2019b; Zhao et al., 2021). The aim of this study was to investigate the inhibitory effect of AEW on two kinds of pathogenic fungi. We found that the susceptible index of citrus fruits decreased obviously. The results showed that the surface of mycelium collapsed, twisted and contracted after AEW treatment, which indicated that the content of cell contents decreased significantly and the contents of cell contents leaked out. Similar studies had shown that its effective chlorine component damages the fungal cell membrane structure, causing intracellular substance leakage and deoxyribonucleic acid denaturation (Han et al., 2017; Liao et al., 2017; Shi et al., 2020; Zhang et al., 2023).

Abnormal increase of cell membrane permeability is the early manifestation of cell injury. The cell membrane plays an important role in maintaining the contents, such as sugars, proteins, inorganic salts, which are crucial for cell activities and survival. Damage to the cell membrane can lead to leakage of cellular contents. This study demonstrated that the electrical conductivity of AEW-treated group was higher than that of the control group, which indicated that AEW could increase the permeability of the cell membrane of pathogenic fungi, thus increasing the extracellular electrical conductivity of pathogenic fungi. This was consistent with the results of Ouyang et al. The increase in the characteristic nucleic acid wavelength of 260 nm normally suggest the leakage of nucleic acid and protein, which cause irreversible damage to cell membrane and cell. A_260_ in the treatment group was significantly higher than that in the control group, which indicated that increased leakage of nucleic acid and other substances in the cells of pathogenic fungi, suggesting that the genetic process in the pathogenic fungal cells was greatly affected by AEW pH controls DNA transcription, protein synthesis and enzyme activity. This was consistent with the results of the experiment (Oonmetta-aree et al., 2006). This study demonstrated that AEW treatment increased the extracellular pH of pathogenic fungi, which caused decreased intracellular pH and acidification of cells by proton accumulation, resulting in cytoplasmic acidification, denaturation, and energy loss. The results indicated that AEW could disrupt the cell membrane integrity of *P. citrinum* and *A. sydowii*, resulting in intracellular components leakage and perturbation of the intracellular environment, leading to accelerated cell death of the pathogens. There were many similar studies, such as AEW treatment at pH of 2.82, ACC of 103 μg/mL significantly reduced the number of *Clostridium sakazakii* in fruits and vegetables (Santo et al., 2016), AEW treatment at ACC of 50 μg/mL could inhibit the invasion of strawberry by *Botrytis cinerea* (Beatriz et al., 2019), AEW treatment at pH of 2.8, ACC of 48 μg/mL could inhibit postharvest *Cladosporium* and *Alternaria* diseases of blueberry (Chen, et al., 2019a, 2019b), and AEW treatment at pH of 2.5, ACC of 80 μg/mL could effectively control the infection of *Phomopsis longanae* to longan fruit (Chen et al., 2017). Our future studies will seek analyze the defense mechanism, antioxidant response, and disease-resistant gene expression of tangelo fruits induced with AEW.

## Conclusion

Two strains were successfully isolated from the diseased tangelo fruits and identified through morphological and molecular characterization and pathogenicity assays. Gene fragments of the two strains were amplified using primers ITS1 and ITS4, and sequencing results revealed the highest homology with *P. citrinum* and *A. sydowii*. The results provide a theoretical basis for investigating the occurrence and development of fruit rot in tangelos as well as the diversity of pathogenic microorganisms and the storage characteristics in tangelos during postharvest storage.

AEW is a potential bacteriostatic agent. In-house toxicity testing indicated that AEW could effectively inhibit *P. citrinum* and *A. sydowii*, with EC_50_ values of 85.4 μg/mL and 60.12 μg/mL, respectively. In addition, AEW treatment induced bending and shrinking on the hypha surface, which implied that the contents of the cells were significantly reduced through leakage of the intracellular substances of the mycelium. AEW could accelerate the death of *P. citrinum* and *A. sydowii* by altering the permeability of cell membrane, increasing the pH cell, and destroying the living environment of cells. In view of the problems such as the blindness of using drugs, pollution by chemical fungicides to environment and the resistance of pathogenic bacteria to drugs, AEW present a novel approach for post-harvest tangelo preservation with great prospects for commercial application.

## Data Availability

The datasets generated during and/or analyzed during the current study are available from the corresponding author on a reasonable request.
